# Serological and molecular detection of *Toxoplasma gondii* in terrestrial and marine wildlife harvested for food in Nunavik, Canada

**DOI:** 10.1186/s13071-019-3408-9

**Published:** 2019-04-03

**Authors:** Nicholas Bachand, André Ravel, Patrick Leighton, Craig Stephen, Momar Ndao, Ellen Avard, Emily Jenkins

**Affiliations:** 1 Department of Veterinary Microbiology, Western College of Veterinary Medicine, University of Saskatchewan, Saskatoon, S7H 5B4 Canada; 2 Groupe de Recherche en épidémiologie des Zoonoses et Santé Publique, Département de Pathologie et Microbiologie, Faculty of Veterinary Medicine, Université de Montréal, Saint-Hyacinthe, J2S 2M2 Canada; 3 Canadian Wildlife Health Cooperative, Western College of Veterinary Medicine, University of Saskatchewan, Saskatoon, S7N 5B4 Canada; 4 National Reference Centre for Parasitology, J.D. MacLean Tropical Diseases Centre, McGill University, Montréal, QC H4A 3J1 Canada; 5 Nunavik Research Center, Makivik Corporation, Kuujjuaq, Canada

**Keywords:** *Toxoplasma gondii*, Zoonosis, Food-borne pathogen, Wildlife, Public health

## Abstract

**Background:**

*Toxoplasma gondii,* a zoonotic protozoan parasite, infects mammals and birds worldwide. Infection in humans is often asymptomatic, though illnesses can occur in immunocompromised hosts and the fetuses of susceptible women infected during pregnancy. In Nunavik, Canada, 60% of the Inuit population has measurable antibodies against *T. gondii.* Handling and consumption of wildlife have been identified as risk factors for exposure. Serological evidence of exposure has been reported for wildlife in Nunavik; however, *T. gondii* has not been detected in wildlife tissues commonly consumed by Inuit.

**Methods:**

We used a magnetic capture DNA extraction and real-time PCR protocol to extract and amplify *T. gondii* DNA from large quantities of tissues (up to 100 g) of 441 individual animals in Nunavik: 166 ptarmigan (*Lagopus lagopus*), 156 geese (*Branta canadensis* and *Chen caerulescens*), 61 ringed seals (*Pusa hispida)*, 31 caribou (*Rangifer tarandus*) and 27 walruses (*Odobenus rosmarus*).

**Results:**

DNA from *T. gondii* was detected in 9% (95% CI: 3–15%) of geese from four communities in western and southern Nunavik, but DNA was not detected in other wildlife species including 20% (95% CI: 12–31%) of ringed seals and 26% (95% CI: 14–43%) of caribou positive on a commercial modified agglutination test (MAT) using thawed heart muscle juice. In geese, tissue parasite burden was highest in heart, followed by brain, breast muscle, liver and gizzard. Serological results did not correlate well with tissue infection status for any wildlife species.

**Conclusions:**

To our knowledge, this is the first report on the detection, quantification, and characterization of DNA of *T. gondii* (clonal lineage II in one goose) from wildlife harvested for food in Nunavik, which supports the hypothesis that migratory geese can carry *T. gondii* into Nunavik where feline definitive hosts are rare. This study suggests that direct detection methods may be useful for detection of *T. gondii* in wildlife harvested for human consumption and provides data needed for a quantitative exposure assessment that will determine the risk of *T. gondii* exposure for Inuit who harvest and consume geese in Nunavik.

## Background

Harvested wildlife is an important source of “country food” in the Canadian North [1, 2]. Country foods include terrestrial/marine mammals, land/sea birds, fish, plants and berries harvested as food from the local natural environment [3]. Consumption of food products of wildlife origin is frequent in Nunavik, northeastern Canada where it contributes up to 25% of people’s daily protein requirements and is consumed at least five times weekly year-round [4]. Although country food is beneficial nutritionally and for ensuring food security, it can harbor chemical, physical and biological hazards sometimes harmful to human health including food-borne zoonotic parasites [5]. In the Arctic, Inuit are potentially exposed to a range of pathogens through frequent subsistence hunting and consumption of raw or undercooked animal tissues from different wildlife species [6]. Not all food-borne hazards can be observed grossly through visual inspections undertaken by hunters or even during controlled, systematic meat inspection [7]. Understanding which zoonotic pathogens are found within wildlife reservoirs in the North is, therefore, needed to evaluate health risks for people who rely on the frequent consumption of wildlife.

Toxoplasmosis, a common infection in humans globally, is caused by the zoonotic parasite *Toxoplasma gondii* [8, 9]. Its life cycle involves three distinct infectious life stages: (i) sporozoites contained within oocysts excreted in the feces of its definitive host (felids); (ii) tachyzoites that travel through blood and cross blood barriers (e.g. placental, ocular and brain) in both definitive and intermediate hosts; and (iii) bradyzoites contained within cysts in tissues of definitive and intermediate hosts [10]. This zoonotic parasite can persist lifelong in its hosts as bradyzoites that divide and multiply slowly within tissue cysts that remain latent [11]. This lifelong persistence within animal tissues is a key feature of the epidemiology of *T. gondii* in humans since the parasite can persist through trophic interactions of intermediate hosts (carnivory) without a need for sexual reproduction in the definitive host [9]. In areas where definitive felid hosts are rare to absent and where the viability of oocysts is likely limited by freezing conditions, such as the Canadian Arctic, this could explain how people and animals are exposed to *T. gondii* [12].

One third of the global human population has been exposed to *T. gondii*, compared to 60% of Inuit in Nunavik, Canada [4, 13]. Food-borne transmission is considered an important route of exposure for Inuit since definitive hosts (felids) that shed oocysts are rare to absent north of the treeline [14]. Inuit regularly consume organs and tissues from several wildlife species raw or undercooked, a food preparation method considered as high risk for exposure to viable *T. gondii* tissue cysts [15]. Two studies in Nunavik have identified the consumption and/or handling of different wildlife species [caribou (*Rangifer tarandus*), seals (several species) and feathered game] as important risk factors for Inuit exposure to *T. gondii* [14, 16]. A regional serological screening programme initiated for pregnant women in the early 1980s showed that congenital toxoplasmosis (seroconversion of the mother during pregnancy) was higher in Nunavik compared to the remainder of Canada (1.8% compared to 0.2% respectively) [17]. There is therefore a need to determine whether people are potentially exposed to infected tissues from hunter-harvested wildlife commonly consumed in Nunavik.

Although exposure to *T. gondii* has been serologically demonstrated in over 300 species of mammals and 30 species of birds worldwide [18], including seals, geese and ptarmigan in Nunavik [19], direct detection of DNA or organism in tissues from wildlife is far less common. This is partly because wildlife pathogen investigations in general present unique challenges due to difficulties with accessing freely-roaming wildlife in remote areas, limited local capacity for testing, and diagnostic tests that are often not validated or optimized for use in wildlife [20]. Most studies in animals rely on detection of antibodies in blood, but this reflects lifetime exposure to, rather than active infection with, *T. gondii*. Because blood or serum is rarely accessible from carcasses of hunter-harvested wildlife, detection of antibodies to *T. gondii* in meat fluid has also been proposed as a suitable alternative in large-scale monitoring programs [21–23]. However, relying on serology as a food safety screening test in wildlife could lead to the rejection of seropositive animals that are not actively infected, which is undesirable in the North where ensuring food security remains an ongoing challenge [4].

Indirect detection methods for *T. gondii*, such as bioassays, also have limitations [24]. Cat bioassays, the gold standard for *T. gondii* detection, require up to 500 grams of tissue in feeding trials although this also has the advantage of increasing the possibility of detecting a tissue cyst. Moreover, not all strains of *T. gondii* produce clinical disease in every animal model (cat or mouse) since virulence is strain and host specific [12]. Bioassays also have the disadvantage of being time-consuming, costly and requiring high numbers of animals which make the method impractical and unethical for wildlife studies [25]. For these reasons, direct detection methods for DNA of *T. gondii* are increasingly used in food safety settings. However, kit-based DNA extraction methods from small tissue quantities (on the order of 25–100 mg) limit detection since *T. gondii* tissue cysts are not uniformly distributed in tissues [26, 27]. As a result, a magnetic-capture DNA extraction and real-time PCR method (MC-PCR) has been developed for testing up to 100 grams of tissue, allowing for improved detection and quantification of parasite DNA [24, 25, 28, 29]. Briefly, sequence-specific DNA fragments bound to magnetic beads help to capture low concentrations of parasite DNA against high backgrounds of host DNA and inhibitory PCR products [28]. Once concentrated using a magnet, the captured DNA sequences are amplified using a quantitative real-time PCR assay based on a highly conserved and sensitive 529 bp non-coding DNA fragment present in 200–300 copies per *T. gondii* genome [30]. In Europe, the MC-PCR technique has also been used as a screening tool in food production animals for research purposes [31]. Recently, it has been used successfully in naturally-infected foxes of Nunavik [32]. There is clearly a need, and now a good method, to determine whether *T. gondii* DNA is present in tissues of wildlife commonly consumed by Inuit of Nunavik, and to compare these results with serological findings based on a commonly used agglutination assay.

## Methods

### Study design

A cross-sectional study was designed to detect DNA of *T. gondii* in the tissues of migratory geese (*Branta canadensis* and *Chen caerulescens*), willow ptarmigan (*Lagopus lagopus*), and ringed seals (*Pusa hispida*) harvested by local hunters as part of regular subsistence activities in the three following communities of Nunavik, Québec (QC), Canada: Kuujjuaraapik (55°16′28″N, 77°45′49″W), Inujuak (58°45′51″N, 78°10′51″W) and Puvirnituq (60°03′71″N, 77°26′92″W). Hunters were informed of the study by a local community coordinator with consent from the local hunter association. Wildlife samples were submitted on a volunteer basis between April 2015 and September 2016. The target sample size was calculated using prevalence estimates from the literature, a 5% precision level, and a 95% confidence interval as follows: ringed seals (*n* = 104 with expected prevalence of 7.3%); Canada geese (*n* = 140; 35 pools of 4 individuals based on an expected prevalence of 4.2%); and willow ptarmigan (*n* = 95; 19 pools of 5 individuals based on an expected prevalence of 2.5%) [19, 33, 34].

### Tissue samples

Local hunters recorded information on species, sex, harvest location, and date. Tissues collected for each animal varied according to wildlife species: seal kits contained the entire heart, at least 100 g each of diaphragm and liver, and the tongue; goose kits included the head, the heart, the gizzard, the liver and at least 100 grams of breast muscle; and the entire carcass was collected from ptarmigan. Samples were stored at -20 °C for less than 2 months before analysis in the laboratory. Caribou samples (e.g. brain, heart, muscle, and sera) archived since 2013 from the Leaf River Herd in Nunavik by biologists and held at -20 °C until processing in 2016, whereas walrus tongues archived as part of a regional *Trichinella* spp. monitoring program at the Nunavik Research Centre. Authorizations were obtained from a major body representing Inuit of Nunavik, the Makivik Corporation, and the Regional Nunavimmi Umajulivijiit Katujaqatigininga (RNUK) during a regional hunter association meeting in Kangiqsualujjuaq in November 2014. Since animals were harvested for other purposes, this work was considered Category A by the University of Saskatchewan Animal Research Ethics Board.

### *Toxoplasma gondii* serology

Sera were available only for caribou, while for seals, ptarmigan, and geese, whole hearts kept frozen in individual plastic bags were thawed at room temperature and fluid was collected from the bag using a sterile disposable plastic pipette [21]. For each species, a modified agglutination test was used as per manufacturer instructions (MAT, New Life Diagnostic LLC, Carlsbad, CA, United States) with a threshold dilution of 1:25 [35]. We used both positive and negative controls supplied in the commercial kit. Since blood from marine mammals contains lipids that may interfere with the performance of agglutination assays [36], we removed lipid from seal samples using a chloroform method, re-tested using MAT, and compared serological test results with the ID Screen® Toxoplasmosis Indirect Multiple-Species ELISA kit used as per manufacturer instructions (IDVet Innovative Diagnostics, Montpellier). To ensure that lipid removal did not interfere with subsequent analyses using MAT, heart fluid from naturally-infected foxes and sera from experimentally-infected reindeer were included as positive controls, and these remained positive following lipid removal [32, 37]. ELISA results were measured as optical density percentages (OD %) as per manufacture instructions, where an OD% greater than 50% is positive, between 40–50% is ambiguous, and less than 40% is negative.

### Extraction and detection of DNA

DNA was extracted from wildlife tissues as per [28] with a minor modification for avian (brain, heart) and caribou samples less than 25 g, which were instead pooled and digested in 50 ml centrifuge tubes rather than stomacher bags. For seals (heart, liver, diaphragm), walrus (tongue) and goose tissues (breast muscle and liver), up to 100 g of each tissue was weighed to determine the required amount of cell lysis buffer (CLB) based on 2.5 ml CLB per gram of tissue. For geese and ptarmigan, aliquots of digest of brain and heart from five birds were pooled. Digests were incubated overnight followed by homogenization by manual vortexing for one minute. For each heart or brain PCR-positive pool, reserved lysate from individual animals (heart and brain) was subsequently analysed separately. Other tissues (liver, gizzard and breast muscle) were analysed for individual geese from PCR-positive brain or heart pools.

Real-time PCR amplification was done in a Bio-Rad CFX 96 DNA thermal cycler (Biorad, Hercules, California, USA) based on a published protocol for the detection of the 188 bp *Toxoplasma* sequence within the 529 repeat-element with the forward primer TOX 9 (5′-agg aga gat atc agg act gta g-3′) and backward primer TOX 11 (5′-gcg tcg tct cgt cta gat cg-3′) as per [28] and [38]. The final PCR assay reaction included 6.5 µl (0.5 M) of Itaq Supermix, 0.25 µl (20 µM) of TP1 probe, 1.25 µl (10 µM) of Tox 9F, 1.25 µl (10 µM) of Tox 11R, 0.5 µl (2 femtograms) of a competitive internal amplification control (CIAC), 1 µl (5 µM) of CIAC probe, 6.75 µl of PCR-grade water and 8 µl of template DNA [32]. A positive PCR reaction was defined as any reaction with a Ct-value smaller or equal to 35, a control negative PCR with a Ct-value of zero, a negative extraction control with a Ct value of zero and a control positive extraction control with a Ct-value smaller or equal to 40 [31]. A negative PCR reaction was defined as a reaction with a Ct-value of zero or above 35, a positive CIAC Ct-value, a negative DNA extraction control with a Ct-value of zero and a positive DNA extraction control with a Ct-value of 40 or less. All reactions for which only one of two replicates amplified, or where CIAC amplification did not occur, were repeated. The DNA from positive PCR products was then purified using the EZ-10 Spin Column PCR Products Purification Kit (Bio Basic, Markham, Ontario) before being sent for DNA sequencing (Macrogen Inc., Korea). DNA sequences were then analyzed using the online Basic Local Alignment Search Tool (BLAST) tool.

### DNA characterization

#### GRA6 DNA extraction

For strongly positive samples (a real-time positive PCR Ct-value < 32), DNA was extracted from 6–12 ml of frozen lysate using 15 pmol of primers targeting the GRA6 gene (GRA6-CapF and GRA6-CapR) rather than 10 pmol [28]. The purified DNA product was sent within 24 h on dry ice to the National Reference Centre for Parasitology, Research Institute of the McGill University Health Centre, Montreal, QC, Canada for further genetic characterization.

#### PCR RFLP amplification

Amplification of the GRA6 gene was done using a published protocol [39]. Briefly, amplification was performed in 50 µl which included 2 µl of DNA template, 5× GoTaq Flexi buffer (Promega), 2 mM MgCl_2_, 50 pmol of each primer, 0.2 mM of each deoxynucleotide triphosphate and 1.25 U of Taq DNA polymerase. Reactions were incubated at 94 °C for 5 min, followed by 35 cycles of denaturing for 30 s at 94 °C, annealing for 60 s at 54 °C, and extension for 90 s at 72 °C. The final cycle was followed by an extension step of 7 min at 72 °C. Two µl of this final PCR product was then used as template DNA in the secondary PCR which used a forward primer (5′- GTA GCG TGC TTG TTG GCG AC-3′) and reverse primer (5′-TAC AAG ACA TAG AGT GCC CC-3′) described by [40] at an annealing temperature of 60 °C and an extension of 2 min with 35 cycles. Five µl of amplicon was run in a 1.5% agarose gel containing GelRed at 120 V for 40 min with 1× TE buffer prior to being visualised under UV light. RFLP analyses were performed on PCR-positive samples in order to characterise the strain type. GRA6 positive amplicons were incubated with the MseI enzyme according to the manufacturer’s instructions (New England BioLabs) and digested PCR amplicons were visualized by electrophoresis on a 1.6% agarose gel containing Gel Red. The unpurified PCR product was sequenced at McGill University and the Génome Québec Innovation Centre in Montreal, QC, Canada. Nucleotide sequences were applied to a BLAST in order to determine % similarity with GRA6 sequences deposited in GenBank.

### Data analysis

#### Prevalence

Seroprevalence and PCR prevalence and their 95% confidence intervals (CI) were estimated using the Ausvet Epitools epidemiological calculators [41].

#### Lowest detection limit and quantification

Determination of the minimum number of DNA copies and tachyzoites detected by the MC-PCR technique has been described elsewhere [32]. Ct-values resulting from the amplified DNA recovered from the spiked beef samples for determining the lowest detection limit were then used to estimate the equation that predicts the log_10_ (concentration) by fitting a generalized linear model in R statistical software version 3.4.4. [28].

#### Serological test agreement (seals)

In seals, proportion of positive results was compared between the MAT and the ELISA using McNemar’s chi-square test. If not significantly different, the kappa coefficient was used to determine the level of agreement between the two tests [33].

## Results

### Wildlife samples

A total of 166 willow ptarmigan, 156 geese, and 61 ringed seals were received. Of the 156 geese, 148 were Canada geese (*Branta canadensis*) and 8 were snow geese (*Chen caerulescens*). A total of 31 caribou sampling kits (16 adult females and 15 calves) from the Nunavik Leaf River herd in 2013, as well as 27 walrus tongues from the Nunavik Research Centre were analyzed. Information on the weight of different tissues analysed for different species is displayed in Table 1.

**Table 1:** Average weight of tissues analysed using the MC-PCR method for detecting *T. gondii* DNA in harvested wildlife from Nunavik, Canada

Species	*n*	Tissues	Average weight (g)	Min. weight (g)	Max. weight (g)
Seal	61	Heart	90.2	32	100
		Liver	74.4	35	100
		Diaphragm	83.4	16	100
Geese	156	Brain	16.3	4.2	26
		Heart	18.4	9.2	26
		Gizzard	72.3	28	100
		Liver	46.2	22	100
		Breast muscle	76.5	36	100
Ptarmigan	166	Brain	7.8	6.7	9.2
		Heart	2.1	0.7	2.9
Caribou	31	Brain	21.4	2.8	91
		Heart/Muscle	52.3	10.7	87
Walrus	27	Tongue	51.4	37	58

### Detection of *T. gondii* antibodies

Antibodies were detected on MAT of heart fluid in 20% of ringed seals (95% CI: 12–31%) and 26% of caribou (95% CI: 14–43%) (Table 2). For geese, seroprevalence was estimated at 11% (95% CI: 6–17%) and 2 of the 18 seropositive geese were snow geese (Table 3). No detection occurred for ptarmigan (Table 1), and serological testing was not possible for walruses. Following lipid removal, no seals were positive on MAT, and positive controls remained positive. Using the ELISA, seroprevalence in seals was estimated at 30% (95% CI: 20–42%) (Table 2).

**Table 2:** Seroprevalence of *T. gondii* in hunter-harvested wildlife in Nunavik, Canada

Species	*n*	Seroprevalence (95% CI) [No. of positive/Total no. analyzed]	
		MAT	ELISA
Ptarmigan	166	0%	np
Caribou	31	26% (14–43%) [8/31]	np
Adult females	16	31% (14–56%) [5/16]	np
Calves	15	20% (7–45%) [3/15]	np
Ringed seals	61	20% (11–31%) [12/61]	30% (20–42%) [18/61]

*Abbreviations*: np, not performed; CI, confidence interval

**Table 3:** Prevalence of *T. gondii* based on the modified agglutination test (MAT) and the magnetic capture and real-time PCR technique in migratory geese harvested in Nunavik, Canada

Species	*n*	Seroprevalence (95% CI)	MC-PCR (95% CI) [No. of positive/total no. analyzed]					
		Heart fluid	Brain (B)^a^	Heart (H)^a^	H or B^b^	Liver	Muscle	Gizzard
Geese	156	11% (7–18%)	4% (0–8%) [9/41]	4% (0–8%) [9/41]	9% (3–15%) [13/41]	14% – [1/7]	31% – [4/13]	9% – [1/11]

^a^Pools were constituted of 5 individual brains or hearts and “n” is the number of pools

^b^PCR-prevalence based on whether pools were positive on brain, heart or both

### Lowest detection limits and quantification

The estimated 95% lowest detection limit for the MC-PCR technique was 445 tachyzoites per 100 g (95% CI: 86–742,000) [32]. For quantification, a generalized linear model was fitted using Ct-values generated from the lowest detection limit experiments with known tachyzoite concentrations used to spiked 100-g beef muscle samples. The best fitting model was described by Ct = 43.3–3.07 log_10_ (tachyzoite) with the outcome being the number of tachyzoite-equivalents per 100 g of tissue. The linear regression showed that the Ct-value could statistically significantly be predicted by the log (tachyzoite) with *F*_(1.92)_ = 2172, *P* < 0.005. The log [tachyzoite] accounted for 96% of the explained variability in the Ct-value. The intercept with the y-axis was 43.3 (95% CI: 42.6–43.9) and the slope was -3.07 (95% CI: -3.2– -2.9). This formula was rewritten as log_10_ (tachyzoites) = (43.3–Ct)/3.07) to more simply estimate the number of tachyzoite-equivalents from the Ct-value in field samples.

### Detection of *T. gondii* DNA from samples

DNA of *T. gondii* was detected in 9% (CI: 3–15%) of geese (Table 3) and no detection occurred for any other wildlife species including ringed seals and caribou positive on serology.

### Genotyping using the GRA6 gene

Out of 5 goose samples with a qPCR Ct-value between 30–33, only one amplified on PCR using primers for the GRA6 gene. On PCR-RFLP, this was identified as the Type II clonal lineage of *T. gondii* which was confirmed by sequencing.

### Parasite burden in geese tissues

Based on the log_10_ (tachyzoites) = (43.3–Ct)/3.07) equation and Ct-values obtained from field samples, the parasite burden defined as the mean number of tachyzoite-equivalents per gram (TE/g) for each tissue and its standard error was: 744 (SE: 476) for heart (*n* = 8), 300 (SE: 100) for brain (*n* = 9), 104 (SE: 140) for breast muscle (*n* = 4), 33 for liver (*n* = 1) and 8 for gizzard (*n* = 1).

### Agreement between serological tests (seals)

In total, heart fluid was available for comparison between the MAT and ELISA for 55 seals:2 seals were positive on both, 10 samples were positive for MAT but negative for ELISA, 16 were negative for MAT but positive for ELISA, and 27 were negative on both. The McNemar’s chi square test comparing the MAT and ELISA serological assays was significant (*P* = 0.029), meaning that there was a difference between results from both serological assays in the case of seals. Therefore, a kappa test statistic was not performed [33].

## Discussion

We directly detected DNA of *T. gondii* in multiple tissues of naturally-infected geese harvested for food by local hunters of Nunavik. This supports previous epidemiological associations between consuming waterfowl and Inuit exposure to *T. gondii* in Nunavik, where the average regional human seroprevalence is 60% [14]. This also shows that migratory geese carry *T. gondii* between southern and northern ecosystems [42, 43], which is further supported by detection of *T. gondii* on mouse and cat bioassays of heart digests from Canada geese in Maryland, USA [44], and brain digests from four Canada geese in Mississippi, USA [45]. The MC-PCR technique used in the current study is specific for *T. gondii* as it targets the highly conserved 529 repeat-element absent in other coccidian parasites such as *Sarcocystis* and *Neospora* spp. [28], and is highly sensitive since there are 200–300 copies per *T. gondii* genome [30]. Moreover, this technique uses large amounts of tissue (up to 100 g) which increases the probability of including a portion of tissue containing parasite DNA. Nonetheless, prevalence based on direct detection has likely been underestimated because levels of parasites in tissues of naturally-infected wildlife may be below the detection limit of the MC-PCR technique used in this study and because *T. gondii* cysts are not uniformly distributed among and within tissues of infected animals [12].

DNA of *T. gondii* was detected in several goose tissues destined for human consumption including heart, liver, gizzard, and breast muscle. This suggests that consumption of infected undercooked geese can lead to food-borne transmission of *T. gondii* in Nunavik [13]. Tissue burdens (number of bradyzoites per gram of tissue) found in this study could be high enough to produce infection given daily goose consumptions trends by Inuit throughout Nunavik (between 0.1–0.3 g per kg body weight daily depending on the region) [46] and using infectious doses for experimentally infected cats (10 bradyzoites), in the absence of data for humans [47]. These findings may have public health implications for Inuit who consume goose tissues raw and undercooked since these may be infected with viable *T. gondii* bradyzoites. Future work includes an exposure assessment for estimating the risk of human exposure to *T. gondii* in Nunavik through goose consumption, a more thorough assessment of the infection status of goose tissues other than heart and brain, as well as an assessment of *T. gondii* viability in country foods prepared in traditional ways.

The *T. gondii* strain detected in one goose in this study was characterized as Type II based on the GRA6 gene. This is one of the three main clonal lineages recognised in North America, where Type II strains are responsible for the majority of congenital infections and infections in people with AIDS [48]. However, because most genetic markers distinguish two of the three clonal lineages, using a single marker can limit the ability to detect non-clonal strains [49]. Characterization results in this study should therefore be interpreted with caution despite the fact that the GRA6 gene is reported to differentiate among the three main clonal lineages [40]. Recent studies have demonstrated the occurrence of atypical strains in North American wildlife including geese [44, 50]. Further studies are therefore needed to better characterize genetic diversity of *T. gondii* in geese harvested in Nunavik.

Evaluating serology against direct detection was an important objective of the paper from a food safety perspective, since detection of antibodies to *T. gondii* in meat juice has been suggested to be a good screening tool in animals slaughtered for human consumption [21]. We chose to use MAT since it has been widely used to detect antibodies to *T. gondii* in sera from caribou, geese, ptarmigan, seals and walrus [19, 34, 37, 51–54]. Recently it has been used to detect antibodies in meat juice from pigs, rabbits, and sheep [21, 55, 56]. In experimentally infected pigs, there was a strong correlation (*r* = 0.87; *P* ˂ 0.001) between detection of antibodies in serum and meat juice from heart [56]. Heart fluid was used in the current study since it was not possible to obtain serum from hunter-harvested wildlife, and to determine if antibody detection in heart fluid could be a useful screening test in the field.

Results from this study demonstrated frequent discrepancies between serological and molecular results in both directions (e.g. seronegative animals with positive tissues, and seropositive animals with absent detection in tissues) which could be accounted for by biological reasons, such as waning of antibodies in individuals with chronic infections. In acute infection, it is possible that tissue invasion has not yet occurred despite the occurrence of detectable antibodies. In another study, one cat inoculated with heart digest from four seronegative geese excreted viable *T. gondii* oocysts, which also suggests that serological status is not a reliable indicator of infection status in this species [44]. In addition to biological reasons for these discrepancies, there are a number of sampling, handling, and diagnostic test characteristics that may play a role. In our study, the cut-off value of a 1:25 dilution on the MAT was used to differentiate seronegative from seropositive geese, but it is possible that antibody levels were too low to be detected, leading to the classification of false-negative geese on serology. Dilutions lower than 1:25 (such as 1:5) might be more sensitive, but could also lead to false-positive results [57]. It is also possible that the high blood content in the heart juice interfered with antibody binding, which has been observed with ELISA in rabbit meat cuts [55]. Freezing and thawing could also affect sample quality though this has not been reported to compromise the detection of antibodies even after 120 days of freezing [55]. Cross-contamination of *T. gondii* DNA between samples could have occurred (leading to false-positive results on tissue testing), but negative controls remained negative.

Our results suggest that MAT and ELISA assays commonly used as screening tools for exposure to *T. gondii* in marine mammals should be interpreted cautiously. Our seroprevalence estimates in ringed seal (MAT 20%, ELISA 30%) were comparable to previous estimates of 7–14% in seals in Nunavik based on a MAT [19, 34]. However, no *T. gondii* DNA was detected in any of the seal tissues analysed in our study, and all seals became seronegative on MAT after lipid removal. To ensure that lipid or other factors in seal tissues do not interfere with the extraction and subsequent detection of DNA using the magnetic capture method, three different types of seal tissues used (heart, liver, diaphragm) were spiked with different tachyzoite concentrations and DNA was detected in all cases (data not shown in this study). The absence of detection of DNA should be interpreted with caution since our sample size (*n* = 61) was small and likely did not confer enough power to detect *T. gondii* DNA in ringed seals if it is present at low prevalence and intensity. While it is possible that tissue burdens in seals may simply be below the detection level of the MC-PCR technique used in this study, this suggests a need to validate the use of serological assays for antibody to *T. gondii* in marine mammals and to carefully interpret previously published findings. Moreover, serological assays, while useful for obtaining a snapshot of exposure status in wildlife populations, should not be used to make decisions on the possible infection status of tissues from an individual animal (i.e. for food safety decisions or when the decision impacts public health, such as in Nunavik). False-positives on serology, resulting in discarding a healthy animal as a source of food, could compromise food security for individuals who rely on or prefer harvested wildlife, especially in Nunavik where one of four households is considered to be food insecure [4]. More in-depth analyses are therefore necessary to examine the extent to which serology is able to predict parasite presence in tissues.

Similar to seals, we did not detect DNA of *T. gondii* in tissues of walruses. We only had access to archived tongue samples for walruses; *T. gondii* has been detected in tongue in some experimentally-infected species [31]. Future research should include a panel of tissues to determine occurrence and tissue predilection of *T. gondii* in walrus. If tongue proves to be a predilection site, detection of *T. gondii* in tongues of walrus could be added to the currently well-established *Trichinella* monitoring programme at the Nunavik Research Centre [58].

We did not detect *T. gondii* DNA in ptarmigan or caribou, two endemic terrestrial wildlife species of Nunavik. Herbivores are generally infected with *T. gondii via* ingestion of oocysts shed in the environment by felid hosts (rare to absent in Nunavik) or tachyzoites that cross the placenta to infect the fetus when a female is infected for the first time in pregnancy. Ptarmigan was the only species in this study which displayed consistent negative serological and molecular results. One study previously reported a *T. gondii* seroprevalence (using MAT) of 2.5% in 70 ptarmigan from communities in Ungava Bay [19], whereas ptarmigan in the current study originated from Hudson Bay. Imperfect test performance and the use of different media (sera *vs* tissue fluid) to detect antibodies may also explain these differences. Although MAT has recently been validated for use in chickens, test performance was shown to be poor [57]. At the moment, there is little evidence of exposure to or infection with *T. gondii* in ptarmigan of Nunavik, which supports the hypotheses that oocyst transmission is rare in northern ecosystems and that ptarmigan represent a low food safety concern with respect to *T. gondii*.

No DNA of *T. gondii* was detected in caribou tissues (muscle, heart, brain) from Nunavik’s Leaf River herd despite detection of *T. gondii* antibodies in the sera of 23% of 30 caribou. This is much higher than the previously reported *T. gondii* seroprevalence of 1.5% (*n* = 268) using another MAT, but lower than the 62.5% (*n* = 40) based on the Sabin-Feldman dye test reported in Kuujjuaq [16, 19]. Very few studies have attempted to correlate the serological status of an animal with the presence of *T. gondii* in their tissues [21]. In domestic animals, a correlation between serological and tissue infection status has been reported in pig and sheep, but not in cattle [59]. In our study, four caribou calves were seropositive, which could be due to transfer of maternal antibodies (one had a seropositive dam), congenital transmission of the parasite, or infection *via* oocyst consumption. For caribou, only small portions of each tissue (muscle, heart and brain) were available for DNA isolation which limited detection probability compared to avian species where whole organs where analyzed. Our seropositive samples, in combination with findings of DNA in all tissues examined in reindeer experimentally exposed to high doses of Type III *T. gondii* oocysts [37], suggest that further work is needed to determine the tissue infection status of naturally-exposed caribou. Future research should use large amounts (at least 100 g) for different caribou tissues in order to provide more insight on the food safety risk of *T. gondii*.

## Conclusions

Detection of *T. gondii* DNA in several goose tissues commonly consumed by people may partially explain the high levels of *T. gondii* exposure observed in Nunavik, Canada. However, since both *T. gondii* prevalence and consumption trends (preparation method, consumption frequency) affect the risk of exposure to *T. gondii*, a better understanding of goose consumption trends in Inuit and an exposure assessment are needed to better answer this question. Since serological and molecular results were often discordant, generally biased towards higher seroprevalence than tissue prevalence, our work suggests caution in using serology as a means of screening positive animals as a food safety prevention measure against a backdrop of food insecurity. Also, future research on other wildlife species endemic to Nunavik should aim for higher sample numbers using larger tissue samples (e.g. in caribou and walrus). Finally, because DNA of *T. gondii* was not detected in any terrestrial or marine wildlife species endemic to Nunavik, these results suggest that exposure to *T. gondii* oocysts shed by felids may be less important than food-borne and vertical routes of exposure in the Canadian North.

## Abbreviations

BLAST: basic local alignment search tool
CI: confidence interval
CIAC: competitive internal amplification control
CLB: cell lysis buffer
Ct: cycle threshold
ELISA: enzyme-linked immunosorbent assay
MAT: modified agglutination test
MC-PCR: magnetic capture and real-time PCR
OD: optical density
PCR: polymerase chain reaction
qPCR: quantitative PCR
RFLP: restriction fragment length polymorphism
RNUK: Regional Nunavimmi Umajulivijiit Katujaqatigininga
